# Supplementary material for: Multi‐dataset identification of innovative feature genes and molecular mechanisms in keratoconus
Source: J Cell Mol Med. 2024 Sep 19;28(18):e70079. doi: 10.1111/jcmm.70079 (PMC11412914; doi:10.1111/jcmm.70079)
Supplement: Supplementary file 2 — Material S2. [file JCMM-28-e70079-s002.zip › 7.GSEA analysis/6.SNORA74B/KEGG_PEROXISOME.html]

Details for gene set KEGG\_PEROXISOME[GSEA]

|  || Dataset | SNORA74B.group.cls#h\_versus\_l |
| Phenotype | group.cls#h\_versus\_l |
| Upregulated in class | h |
| GeneSet | KEGG\_PEROXISOME |
| Enrichment Score (ES) | 0.14234607 |
| Normalized Enrichment Score (NES) | 0.5728001 |
| Nominal p-value | 0.9527559 |
| FDR q-value | 1.0 |
| FWER p-Value | 1.0 |
Table: GSEA Results Summary

  

Fig 1: Enrichment plot: KEGG\_PEROXISOME      
 Profile of the Running ES Score & Positions of GeneSet Members on the Rank Ordered List

  

| SYMBOL | TITLE | RANK IN GENE LIST | RANK METRIC SCORE | RUNNING ES | CORE ENRICHMENT || 1 | FAR2 | na | 694 | 0.545 | 0.0445 | Yes |
| 2 | HAO1 | na | 1078 | 0.475 | 0.0897 | Yes |
| 3 | BAAT | na | 2571 | 0.328 | 0.0854 | Yes |
| 4 | PEX11G | na | 2959 | 0.303 | 0.1101 | Yes |
| 5 | DDO | na | 3063 | 0.298 | 0.1423 | Yes |
| 6 | HMGCL | na | 6817 | 0.168 | 0.0539 | No |
| 7 | CRAT | na | 6977 | 0.163 | 0.0686 | No |
| 8 | ABCD4 | na | 7664 | 0.144 | 0.0659 | No |
| 9 | ACAA1 | na | 7836 | 0.140 | 0.0775 | No |
| 10 | NUDT19 | na | 7964 | 0.137 | 0.0901 | No |
| 11 | PEX11A | na | 8130 | 0.133 | 0.1010 | No |
| 12 | MVK | na | 8473 | 0.125 | 0.1059 | No |
| 13 | ACOX1 | na | 9079 | 0.112 | 0.1017 | No |
| 14 | PEX26 | na | 9781 | 0.097 | 0.0929 | No |
| 15 | FAR1 | na | 11133 | 0.073 | 0.0625 | No |
| 16 | PXMP4 | na | 11219 | 0.071 | 0.0685 | No |
| 17 | ECH1 | na | 11352 | 0.069 | 0.0729 | No |
| 18 | ACOX3 | na | 11425 | 0.068 | 0.0789 | No |
| 19 | ACSL1 | na | 11987 | 0.060 | 0.0698 | No |
| 20 | EPHX2 | na | 12228 | 0.057 | 0.0696 | No |
| 21 | NUDT12 | na | 12525 | 0.053 | 0.0673 | No |
| 22 | PEX16 | na | 12731 | 0.050 | 0.0673 | No |
| 23 | CAT | na | 12780 | 0.050 | 0.0718 | No |
| 24 | PEX12 | na | 12975 | 0.047 | 0.0718 | No |
| 25 | PEX1 | na | 13146 | 0.045 | 0.0722 | No |
| 26 | PMVK | na | 13166 | 0.045 | 0.0770 | No |
| 27 | SCP2 | na | 13399 | 0.043 | 0.0754 | No |
| 28 | GNPAT | na | 14286 | 0.033 | 0.0537 | No |
| 29 | XDH | na | 14497 | 0.030 | 0.0512 | No |
| 30 | PAOX | na | 15281 | 0.022 | 0.0312 | No |
| 31 | SLC27A2 | na | 15285 | 0.022 | 0.0337 | No |
| 32 | MPV17 | na | 15402 | 0.020 | 0.0327 | No |
| 33 | ABCD1 | na | 15620 | 0.018 | 0.0286 | No |
| 34 | HACL1 | na | 15666 | 0.018 | 0.0295 | No |
| 35 | AGPS | na | 16078 | 0.014 | 0.0192 | No |
| 36 | PEX7 | na | 16398 | 0.010 | 0.0113 | No |
| 37 | PHYH | na | 16791 | 0.007 | 0.0007 | No |
| 38 | PEX11B | na | 17331 | 0.001 | -0.0147 | No |
| 39 | PRDX1 | na | 17443 | 0.000 | -0.0179 | No |
| 40 | PECR | na | 17523 | -0.001 | -0.0200 | No |
| 41 | PRDX5 | na | 17569 | -0.001 | -0.0212 | No |
| 42 | PEX10 | na | 17611 | -0.002 | -0.0222 | No |
| 43 | GSTK1 | na | 17937 | -0.005 | -0.0309 | No |
| 44 | PEX14 | na | 19095 | -0.018 | -0.0621 | No |
| 45 | HSD17B4 | na | 19313 | -0.021 | -0.0659 | No |
| 46 | ABCD3 | na | 19705 | -0.025 | -0.0742 | No |
| 47 | ACOT8 | na | 19935 | -0.028 | -0.0775 | No |
| 48 | ECI2 | na | 20064 | -0.029 | -0.0778 | No |
| 49 | PEX19 | na | 20159 | -0.030 | -0.0770 | No |
| 50 | SOD1 | na | 21117 | -0.041 | -0.0997 | No |
| 51 | DHRS4 | na | 21290 | -0.043 | -0.0995 | No |
| 52 | PIPOX | na | 21698 | -0.048 | -0.1056 | No |
| 53 | PEX3 | na | 21818 | -0.050 | -0.1031 | No |
| 54 | PEX13 | na | 22208 | -0.054 | -0.1080 | No |
| 55 | IDH2 | na | 22633 | -0.060 | -0.1131 | No |
| 56 | SLC25A17 | na | 22716 | -0.061 | -0.1082 | No |
| 57 | ACSL4 | na | 23152 | -0.068 | -0.1128 | No |
| 58 | PEX2 | na | 23483 | -0.072 | -0.1138 | No |
| 59 | PEX5 | na | 23851 | -0.078 | -0.1151 | No |
| 60 | DAO | na | 24207 | -0.084 | -0.1154 | No |
| 61 | ACSL6 | na | 24238 | -0.084 | -0.1062 | No |
| 62 | SOD2 | na | 24371 | -0.087 | -0.0998 | No |
| 63 | MLYCD | na | 24915 | -0.097 | -0.1040 | No |
| 64 | IDH1 | na | 25567 | -0.109 | -0.1099 | No |
| 65 | AMACR | na | 26108 | -0.119 | -0.1114 | No |
| 66 | DECR2 | na | 26175 | -0.120 | -0.0991 | No |
| 67 | ACSL3 | na | 26206 | -0.121 | -0.0856 | No |
| 68 | EHHADH | na | 26637 | -0.131 | -0.0825 | No |
| 69 | PEX6 | na | 26757 | -0.134 | -0.0701 | No |
| 70 | CROT | na | 28329 | -0.174 | -0.0948 | No |
| 71 | MPV17L | na | 28469 | -0.179 | -0.0777 | No |
| 72 | ACSL5 | na | 28542 | -0.181 | -0.0584 | No |
| 73 | PXMP2 | na | 29236 | -0.202 | -0.0545 | No |
| 74 | AGXT | na | 29339 | -0.204 | -0.0333 | No |
| 75 | HAO2 | na | 30842 | -0.260 | -0.0459 | No |
| 76 | ACOX2 | na | 31557 | -0.298 | -0.0312 | No |
| 77 | ABCD2 | na | 33578 | -0.460 | -0.0351 | No |
| 78 | NOS2 | na | 34182 | -0.577 | 0.0158 | No |
Table: GSEA details [plain text format]

  

Fig 2: KEGG\_PEROXISOME      
 Blue-Pink O' Gram in the Space of the Analyzed GeneSet

  

Fig 3: KEGG\_PEROXISOME: Random ES distribution      
 Gene set null distribution of ES for **KEGG\_PEROXISOME**
